# Sequential Loss of LC Noradrenergic and Dopaminergic Neurons Results in a Correlation of Dopaminergic Neuronal Number to Striatal Dopamine Concentration

**DOI:** 10.3389/fphar.2012.00184

**Published:** 2012-10-22

**Authors:** Patricia Szot, Allyn Franklin, Carl Sikkema, Charles W. Wilkinson, Murray A. Raskind

**Affiliations:** ^1^Mental Illness Research, Education and Clinical Center, Veterans Administration Puget Sound Health Care SystemSeattle, WA, USA; ^2^Department of Psychiatry and Behavioral Sciences, University of WashingtonSeattle, WA, USA; ^3^Geriatric Research, Education and Clinical Center, Veterans Administration Puget Sound SystemSeattle, WA, USA

**Keywords:** 6-hydroxydopamine, MPTP, locus coeruleus, substantia nigra, ventral tegmentum area, catecholamines, striatum

## Abstract

Noradrenergic neurons in the locus coeruleus (LC) are significantly reduced in Parkinson’s disease (PD) and the LC exhibits neuropathological changes early in the disease process. It has been suggested that a loss of LC neurons can enhance the susceptibility of dopaminergic neurons to damage. To determine if LC noradrenergic innervation protects dopaminergic neurons from damage, the dopaminergic neurotoxin 1-methyl-4-phenyl-1,2,3,6-tetrahydropyridine (MPTP) was administered to adult male C57Bl/6 mice 3 days after bilateral LC administration of 6-hydroxydopamine (6OHDA), a time when there is a significant reduction in LC neuronal number and innervation to forebrain regions. To assess if LC loss can affect dopaminergic loss four groups of animals were studied: control, 6OHDA, MPTP, and 6OHDA + MPTP; animals sacrificed 3 weeks after MPTP administration. The number of dopaminergic neurons in the substantia nigra (SN) and ventral tegmental area (VTA), and noradrenergic neurons in the LC were determined. Catecholamine levels in striatum were measured by high-pressure liquid chromatography. The loss of LC neurons did not affect the number of dopaminergic neurons in the SN and VTA compared to control; however, LC 6OHDA significantly reduced striatal dopamine (DA; 29% reduced) but not norepinephrine (NE) concentration. MPTP significantly reduced SN and VTA neuronal number and DA concentration in the striatum compared to control; however, there was not a correlation of striatal DA concentration with SN or VTA neuronal number. Administration of 6OHDA prior to MPTP did not enhance MPTP-induced damage despite an effect of LC loss on striatal DA concentration. However, the loss of LC neurons before MPTP resulted now in a correlation between SN and VTA neuronal number to striatal DA concentration. These results demonstrate that the loss of either LC or DA neurons can affect the function of each others systems, indicating the importance of both the noradrenergic and dopaminergic system in PD.

## Introduction

Parkinson’s disease (PD) is a neurological disorder that is characterized by various motor deficits including tremor, rigidity, and bradykinesia (Singh et al., 2007). The cause of these motor symptoms is the loss of dopaminergic neurons in the substantia nigra (SN) pars compacta and subsequently reduced dopamine (DA) concentration in the striatum (Gibb, 1991; Gibb and Lees, 1991; Damier et al., 1999). The cause of dopaminergic neuronal loss in PD is still unknown. However, dopaminergic neurons are not the only neurons reduced in PD. Locus coeruleus (LC) noradrenergic neurons are also significantly reduced in PD; the loss of LC neurons is equal to or greater than the loss of dopaminergic neurons in the SN (Cash et al., 1987; Hornykiewicz and Kish, 1987; Chan-Palay and Asan, 1989; Patt and Gerhard, 1993; Bertrand et al., 1997; Zarow et al., 2003; McMillan et al., 2011). Neuropathological changes associated with PD appear in the LC before the appearance in dopaminergic neurons (Braak et al., 2003, 2006). The midbrain dopaminergic neurons in the SN and ventral tegmental area (VTA) receive direct innervation from LC noradrenergic neurons (Swanson and Hartman, 1975; Jones and Moore, 1977; Phillipson, 1979; Simon et al., 1979; Jones and Yang, 1985; Fritschy and Grzanna, 1990; Szot et al., 2012). A reduction in LC noradrenergic activity results in reduced activity of SN and VTA neurons (Grenhoff and Svensson, 1989, 1993; Grenhoff et al., 1993, 1995; Guiard et al., 2008; Wang et al., 2010) and dopamine-induced behavior (Antelman and Caggiula, 1977; Chopin et al., 1999; Rommelfanger et al., 2007; Grimbergen et al., 2009; Taylor et al., 2009; Wang et al., 2010).

If the noradrenergic nervous system experiences a disruption in function before the dopaminergic system in PD (Braak et al., 2003, 2006) and the noradrenergic nervous system directly innervates dopaminergic neurons in the SN and VTA, a loss of noradrenergic neurons could increase the susceptibility of dopaminergic neurons to damage in the progression of PD. There are several studies suggesting that the administration of a noradrenergic neurotoxin prior to the application of a dopaminergic neurotoxin will enhance the susceptibility of dopaminergic neurons to damage (Mavridis et al., 1991; Marien et al., 1993; Bing et al., 1994; Fornai et al., 1995, 1996, 1997; Srinivasan and Schmidt, 2003, 2004; Archer and Fredriksson, 2006). However, these studies are not comprehensive and some used *N*-(2-chloroethyl)-*N*-ethyl-2-bromobenzylamine (DSP4) as the noradrenergic neurotoxin. DSP4 produces a rapid though transient reduction in terminal norepinephrine (NE) concentration in specific forebrain regions (Ross, 1976; Jonsson et al., 1981; Grzanna et al., 1989; Theron et al., 1993; Wolfman et al., 1994; Kask et al., 1997; Hughes and Stanford, 1998; Szot et al., 2010). However, there are data specifically in rats indicating that DSP4 does not result in a loss of LC noradrenergic neurons, suggesting neuronal loss is not to account for the changes observed in noradrenergic forebrain markers (Booze et al., 1988; Lyons et al., 1989; Robertson et al., 1993; Matsukawa et al., 2003; Szot et al., 2010).

Recent work in our laboratory has shown that direct administration of 6-hydroxydopamine (6OHDA) into the LC will specifically reduce LC noradrenergic neurons, resulting in a reduction of NE concentration and NE transporter (NET) in forebrain regions 3 weeks later (Szot et al., 2012). The changes in noradrenergic forebrain markers produced by 6OHDA are different from the changes produced by DSP4 (Szot et al., 2012), suggesting that these two neurotoxins result in different responses of noradrenergic neurons. Therefore, the objective of this study is to determine if the loss of LC noradrenergic neurons induced by LC administration of 6OHDA prior to the administration of the dopaminergic neurotoxin 1-methyl-4-phenyl-1,2,3,6-tetrahydropyridine (MPTP) enhances the susceptibility of dopaminergic neurons in the SN and VTA to damage, and further decrease DA concentration in the striatum. MPTP is administered 3 days after bilateral 6OHDA administration directly into the LC, a time similar to previously published work (Mavridis et al., 1991; Marien et al., 1993; Fornai et al., 1995, 1996, 1997; Srinivasan and Schmidt, 2003, 2004). The number of LC, SN, and VTA neurons is assessed as well as the amount of DA and other catecholamines in the striatum. Correlations of neuronal loss in each treatment group are measured against concentration of catecholamines in the striatum.

## Materials and Methods

### Animals

#### Experiment 1: 3-day 6OHDA study

Thirty adult male C57Bl/6 mice were purchased from Charles River Laboratories (Wilmington, MA, USA) and housed in standard enriched environment cages in a temperature controlled room with a 12-h light/dark cycle. Food and water were provided *ad libitum*. The animals were given at least 2 weeks acclimating period in the facility before administration of 6OHDA. All animal procedures were in accordance with the Animal Care Committee at the VA Puget Sound Health Care System, Seattle, WA, USA, and National Institute of Health guidelines. The minimum number of animals was used for these studies and care was taken to minimize any suffering. Sixteen mice were administered 6OHDA (10 μg/μl) and 14 mice were administered vehicle (0.2% ascorbic acid/saline) *bilaterally* into the LC as previously described (Szot et al., 2010). Animals were sacrificed 3 days later, and brains removed. The hindbrain portion containing the LC and lateral tegmental regions was dissected free and cut on a cryostat at 16 μm onto Superfrost Plus slides (Fisher Scientific, Pittsburgh, PA, USA) into three sets consisting of alternating sections and stored at −80°C. Slides containing the lateral tegmental area had tyrosine hydroxylase (TH)- and dopamine β-hydroxylase (DBH)-*in situ* hybridization (ISH) performed to assess neuronal loss. Slides containing the LC had TH-immunohistochemistry (IHC), TH-, and DBH-ISH performed to assess neuronal loss. Forebrains were either (1) cut as described above (*n* = 8) for NET, α_1_-, and α_2_-adrenoreceptor (AR) binding assays or (2) had the frontal cortex (FC), bed nucleus stria terminalis (BNST)/septum, hippocampus (HP), and amygdala (Amy) dissected free for measurement of catecholamine concentrations by high-pressure liquid chromatography (HPLC; *n* = 6–7).

#### Experiment 2: 6OHDA + MPTP study

Forty-three adult male C57Bl/6 mice were purchased from Charles River Laboratories; 23 mice were administered 6OHDA (10 μg/μl) and 17 mice were administered vehicle (0.2% ascorbic acid/saline) *bilaterally* into the LC. Three days later, 11 6OHDA-treated mice received saline intraperitoneally (IP; two injections 2 h apart; 6OHDA group) while the remaining 15 6OHDA-treated mice received MPTP (24 mg/kg free base, IP; two injections 2 h apart; 6OHDA + MPTP group). The animals that received vehicle into the LC were divided into eight mice receiving saline IP (two injections 2 h apart; control group) and nine mice receiving MPTP (24 mg/kg free base, IP; two injections 2 h apart; MPTP group). Three animals from the 6OHDA + MPTP group and one animal from the MPTP group died within 72 h of the last MPTP injection. Three weeks after MPTP injection animals were sacrificed and brains removed. The striatum was dissected free from the forebrain and catecholamine concentrations were measured unilaterally by HPLC for each animal. Midbrain dopaminergic regions (SN and VTA) and the LC were cut on a cryostat at 16 μm onto Superfrost Plus slides into three sets of slides consisting of alternating sections and stored at −80°C. Slides containing the SN and VTA had TH-IHC and TH-ISH performed to assess neuronal loss. Slides containing the LC had TH-IHC, TH-ISH, and DBH-ISH to assess the neuronal loss.

### TH-IHC

TH-IHC was performed in the SN, VTA, and LC to assess the number of dopaminergic and noradrenergic neurons as previously described with the minor modification of adding 0.2% nickel ammonium sulfate to the DAB visualization step (for SN and VTA regions only; Szot et al., 2012). The number of TH-immunoreactive (IR) cell bodies in the SN, VTA, and LC were counted under 20× magnification in three consecutive atlas matched sections in the 3-day 6OHDA group and in the four groups described above to determine if LC neuronal loss enhanced MPTP-induced damage to dopaminergic neurons. The unilateral number of TH-IR neurons were averaged for each animal and data expressed as TH-IR neurons ± SEM for each group. Photomicrographs were taken with a digital camera and imported into Adobe Photoshop. To optimize visualization of staining, photomicrographs were modified, when necessary, by adjusting brightness and contrast.

### TH- and DBH-ISH

Tyrosine hydroxylase-immunohistochemistry was performed in the SN, VTA, and LC and DBH-ISH in the LC as previously described (Szot et al., 2010, 2012). Briefly, the TH oligonucleotide probe was a 48 base probe complementary to nucleotides 1351–1398 of the rat TH mRNA sequence (Grima et al., 1985). The DBH oligonucleotide probe consisted of two oligonucleotides complementary to nucleotides 454–505 and 1414–1465 of the rat sequence (McMahon et al., 1990). The oligonucleotide probes were 3′ end-labeled with ^33^P-dATP (PerkinElmer, Boston, MA, USA) using terminal deoxyribonucleotidyl transferase (Invitrogen, Piscataway, NJ, USA). The TH probe contained a range from 0.35 to 0.58 × 10^6^ cpm/50 μl for the different experiments and was washed as described in detail in previously published work with the oligonucleotide (Szot et al., 1997). The DBH probe contained a range from 0.5 to 2.9 × 10^6^ cpm/50 μl for the different experiments and washed as described in detail in previously published work with the oligonucleotide (Szot et al., 2010). Slides containing the LC region hybridized with either TH or DBH probes were coated with NTB2 Nuclear Track Emulsion (undiluted; Eastman Kodak Co., Rochester, NY, USA) and stored at −20°C for 4 days for DBH mRNA and 1 week for TH mRNA. Slides containing the SN/VTA region hybridized with TH probe were coated with NTB2 Nuclear Track Emulsion (undiluted; Eastman Kodak Co.) for 1 week. All slides emulsion coated were developed by standard procedures as previously described (Szot et al., 1997).

Quantitation of TH and DBH mRNA expression was similar to that performed and described in previous publications (Szot et al., 2006, 2010) using the MicroComputer Imaging Device System (MCID; InterFocus Imaging, Ltd., Cambridge, England). The number of positive labeled neurons that achieved labeling threefold higher than background was counted bilaterally in all groups across three atlas matched consecutive sections and averaged for each animal. Data for the number of positive labeled neurons for each group were expressed as the average ± SEM. The density of TH and DBH mRNA expression/neuron was performed by measuring the amount of silver grains over cell bodies of labeled neurons that were threefold higher than background under 20× dark-field illumination using MCID and data for grains/neuron were expressed as average ± SEM for each group. All labeled neurons that were counted as positively labeled for each oligonucleotide probe were also quantitated for the amount of TH and DBH mRNA expression/neuron. Photomicrographs were taken with a digital camera and imported into Adobe Photoshop. To optimize visualization of labeling, photomicrographs were modified, when necessary, by adjusting brightness and contrast.

### Receptor binding

^3^H-Nisoxetine (80.0 Ci/mmol; American Radiolabeled Chemicals, St. Louis, MO, USA) was used to quantitate NET binding sites, ^3^H-RX821002 (55.0 Ci/mmol; PerkinElmer) was used to quantitate α_2_-AR binding sites and ^3^H-prazosin (83.6 Ci/mmol; PerkinElmer) was used to quantitate α_1_-AR binding sites. Forebrain AR binding studies were performed only in animals sacrificed 3 days after bilateral LC administration of either vehicle or 6OHDA. ^3^H-Nisoxetine binding was performed as previously described (Weinshenker et al., 2002; Szot et al., 2006, 2010). Briefly, 600 μl/slide of incubation buffer (∼3 nM ^3^H-nisoxetine in 50 mM Tris buffer with 300 mM NaCl and 5 mM KCl, pH 7.7) was placed over the tissue. Non-specific binding was defined in the presence of 1 μM mazindol. Slides were incubated for 2 h at room temperature and then washed twice for 2 min in ice-cold 50 mM Tris buffer, pH 7.4, dipped in ice-cold distilled water to remove salts and then dried rapidly under a stream of cool air. ^3^H-RX821002 binding was performed as described previously (Szot et al., 2006, 2010). Briefly, 600 μl/slide of incubation buffer (∼2 nM ^3^H-RX821002 in 50 mM NaPO_4_ buffer, pH 7.4) was placed over the tissue. Non-specific binding was defined in the presence of 10 μM rauwolscine. Slides were incubated for 45 min at room temperature and then washed for 2 min in ice-cold 50 mM NaPO_4_ buffer, pH 7.4, dipped in ice-cold distilled water and dried as described above for ^3^H-nisoxetine. ^3^H-Prazosin was performed as described previously (Sanders et al., 2006; Szot et al., 2006, 2010). Briefly, 600 μl/slide of incubation buffer (∼0.2 nM ^3^H-prazosin in 50 mM Tris buffer, 1 mM EDTA, pH 7.4) was placed over the tissue. Non-specific binding was defined in the presence of 10 μM phentolamine. Slides were incubated for 40 min at room temperature, washed and dried as described above for ^3^H-nisoxetine. All slides were apposed to Biomax MR Film (Eastman Kodak Co.) for 2 months.

Films were developed by standard procedures (Szot et al., 1997). NET, α_2_- and α_1_-AR binding sites were quantitated as optical density (OD) using MCID system in three consecutive sections atlas matched in control- and 6OHDA-treated groups. NET (^3^H-nisoxetine) binding sites were quantitated (OD) in the following atlas matched regions: FC, septum, BNST, paraventricular thalamic nucleus (PVTN), HP, SN, and VTA. α_2_-AR (^3^H-RX821002) binding sites were quantitated in the following atlas matched regions: FC, septum, BNST, striatum (Str), dorsal thalamic nucleus (DTN), hypothalamus (hypo), HP, Amy, SN, VTA, and geniculate (Gen). α_1_-AR (^3^H-prazosin) binding sites were quantitated in the following atlas matched regions: FC, septum, BNST, thalamus (Thal), HP, Amy, Hypo, SN, and VTA.

Data for NET, α_1_-, and α_2_-AR binding concentrations were expressed as average (OD) ± SEM. Photomicrographs were taken with a digital camera and imported into Adobe Photoshop. To optimize visualization of labeling, all photomicrographs were modified equally, when necessary, by adjusting brightness and contrast.

### HPLC measurement of catecholamines in forebrain regions

The catecholamine precursor 1-3-4-dihydroxyphenylalanine (DOPA), the catecholamines norepinephrine (NE) and dopamine (DA), and the deaminated metabolites of NE and DA dihydroxyphenylglycol (DHPG) and dihydroxyphenylacetic acid (DOPAC) were quantitated in brain tissue extracts by HPLC with electrochemical detection. Each unilateral brain region (FC, BNST/septum, HP, and Amy) collected for Experiment 1 and each unilateral striatal tissue fragment collected for Experiment 2 was sonicated in 0.5 ml of 0.1 M perchloric acid. A 100-μl aliquot of the sonicated material was stored at −80°C for protein determination using Pierce BCA^™^ Protein Assay kit (Thermo Scientific, Rockford, IL, USA). The supernate was collected from centrifugation of the sonicated material at 13,000 × *g* for 15 min and stored at −70°C until catecholamine extraction was performed.

Catecholamines and metabolites were extracted from 100 μl of the sonicated supernate with a modification of the alumina method of Goldstein et al. (1981). The eluted catechols were filtered through a 0.22 Millex^®^GV syringe-driven filter and transferred to an autosampler tube prior to injection. Detection was performed with an ESA Coulochem II electrochemical detector (ESA, Chelmsford, MA, USA) with the conditioning cell set at +350 mV, electrode 1 of the analytical cell set at +90 mM, and electrode 2 of the analytical cell set at −300 mV and a Phenomenex reverse phase c18 Gemini column (150 mm × 4.6 , 3 m, 110 Å; Phenomenex, Torrance, CA, USA). The EZChrom Elite^™^ chromatography data system (Agilent Technologies, Inc., Santa Clara, CA, USA) was used for data reduction. Catecholamine values were expressed as mean ng catecholamine/mg protein ± SEM for each group. All samples from each region were assayed for catecholamines in the same batch in order to reduce variability.

### Statistical analysis

Student’s unpaired *t*-test was used to assess statistical difference using GraphPad Prism for Experiment 1. A one-way ANOVA was used to assess statistical difference followed by a *post hoc* Tukey’s test using the computer program GraphPad Prism (v. 5.0, GraphPad Software, Inc.) for Experiment 2. Simple linear regressions were performed between the number of TH-IR neurons in the LC to the number of TH-IR neurons in the VTA or SN; number of TH-IR neurons in the LC to the concentration of each catecholamine in the striatum; number of TH-IR in the SN to the concentration of each catecholamine in the striatum, and number of TH-IR neurons in the VTA to the concentration of each catecholamine in the striatum.

## Results

### Experiment 1

#### Lateral tegmental regions

Three days after bilateral administration of 6OHDA into the LC, the lateral tegmental area regions, nucleus tract solitaris (NTS), and A1/A2, were unaffected (data not shown). Both TH and DBH mRNA expression data in these regions indicate that administration of 6OHDA directly into the LC does not affect these noradrenergic neurons as demonstrated previously 3 weeks after unilateral LC administration of 6OHDA (Szot et al., 2012).

#### Locus coeruleus

**Figure 1:**
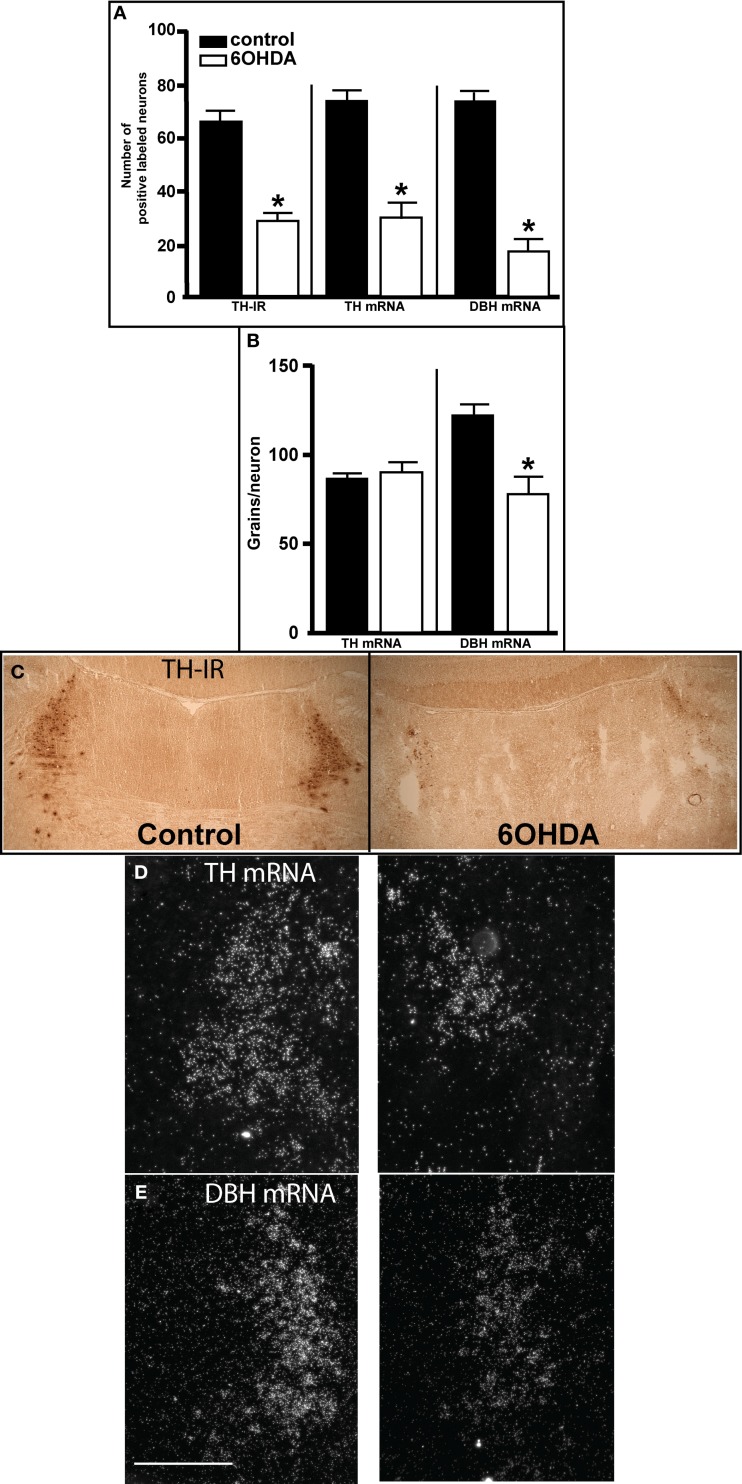
**Assessment of LC noradrenergic neuronal number 3 days after bilateral 6OHDA administration directly into the LC**. **(A)** Quantitation of LC neuronal number by TH-IR, TH-, and DBH mRNA expression, **(B)** quantitation of TH- and DBH mRNA expression/neuron in LC noradrenergic neurons, and photomicrograph of TH-IR **(C)**, TH mRNA **(D)**, and DBH mRNA **(E)** expression 3 days after bilateral vehicle/6OHDA administration. *Indicates significant difference to vehicle. Scale bar = 200 μm.

Three days after bilateral administration of 6OHDA into the LC there was a significant reduction in the number of neurons as observed by TH-IR (56% reduced), TH mRNA (60% reduced), and DBH mRNA (77% reduced) expression (Figures 1A,C–E). The loss of LC neurons as determined by DBH mRNA tended to be greater than the loss measured by TH-IR and TH-ISH (Figure 1A). The enhanced loss of LC neurons as determined by DBH mRNA expression compared to TH mRNA may be due to a 36% decrease in DBH mRNA expression/neuron that was observed 3 days after 6OHDA (Figures 1B,E). TH mRNA expression/neuron was not affected by administration of 6OHD into the LC (Figures 1B,D).

#### Catecholamine concentration in forebrain regions

Bilateral administration of 6OHDA directly into the LC resulted in a significant loss of NE concentration in the FC (31% reduced), HP (21% reduced), and Amy (21% reduced) but not in the BNST/septum compared to vehicle-treated animals (Table 1). In addition, bilateral administration of 6OHDA directly into the LC resulted in a significant loss of the NE metabolite DHPG in all regions studied (FC: 49% reduced; HP: 48% reduced; BNST/septum: 48% reduced; Amy: 47% reduced; Table 1). Bilateral administration of 6OHDA directly into the LC did not affect DOPA, DA, or DOPAC concentration in any of the brain regions studied (Table 1).

**Table 1:** **Catecholamine levels expressed as ng per catecholamine/mg protein in frontal cortex, BNST/septum, hippocampus, and amygdala 3 days after bilateral administration of vehicle and 60HDA**.

	Frontal cortex (FC)		Hippocampus (HP)		BNST/septum		Amygdala (amy)	
	Vehicle	6OHDA	Vehicle	6OHDA	Vehicle	6OHDA	Vehicle	60HDA
DHPG	0.36 + 0.02	0.18 ± 0.03*	0.17 ± 0.02	0.09 ± 0.01*	0.10 ± 0.02	0.05 ± 0.01*	0.27 ± 0.02	0.14 ± 0.02*
NE	5.33 ± 0.26	3.70 ± 0.12*	4.87 ± 0.30	3.83 ± 0.12*	2.40 ± 0.36	1.69 ± 0.13	4.79 + 0.26	3.22 + 0.22*
DOPA	0.05 + 0.004	0.04 ± 0.004	0.09 ± 0.03	0.07 ± 0.007	0.06 ± 0.007	0.08 ± 0.011	0.03 + 0.004	0.03 + 0.003
DA	1.89 ± 0.48	1.12 ± 0.29	0.47 ± 0.08	0.42 ± 0.05	74.9 ± 9.75	82.6 ± 9.14	2.79 ± 0.05	2.93 ± 0.03
DOPAC	1.18 + 0.17	0.95 + 0.13	0.26 + 0.01	0.25 + 0.03	8.65 + 1.12	8.68 + 0.85	0.88 + 0.08	0.90 + 0.05

*Indicates significant difference from vehicle animals.

A correlation between LC neuronal numbers with catecholamine concentrations in forebrain regions was observed for DHPG in the FC (*r*^2^ = 0.41, *P* = 0.023), HP (*r*^2^ = 0.48, *P* = 0.013), and Amy (*r*^2^ = 0.56, *P* = 0.005), but not for NE concentration. The lack of a correlation for NE to LC neuronal number suggests the reduction in NE concentration in the FC, HP, and Amy 3 days after bilateral 6OHDA was less than the loss of LC noradrenergic neurons.

#### NET, α_1_-, and α_2_-AR binding sites

**Figure 2:**
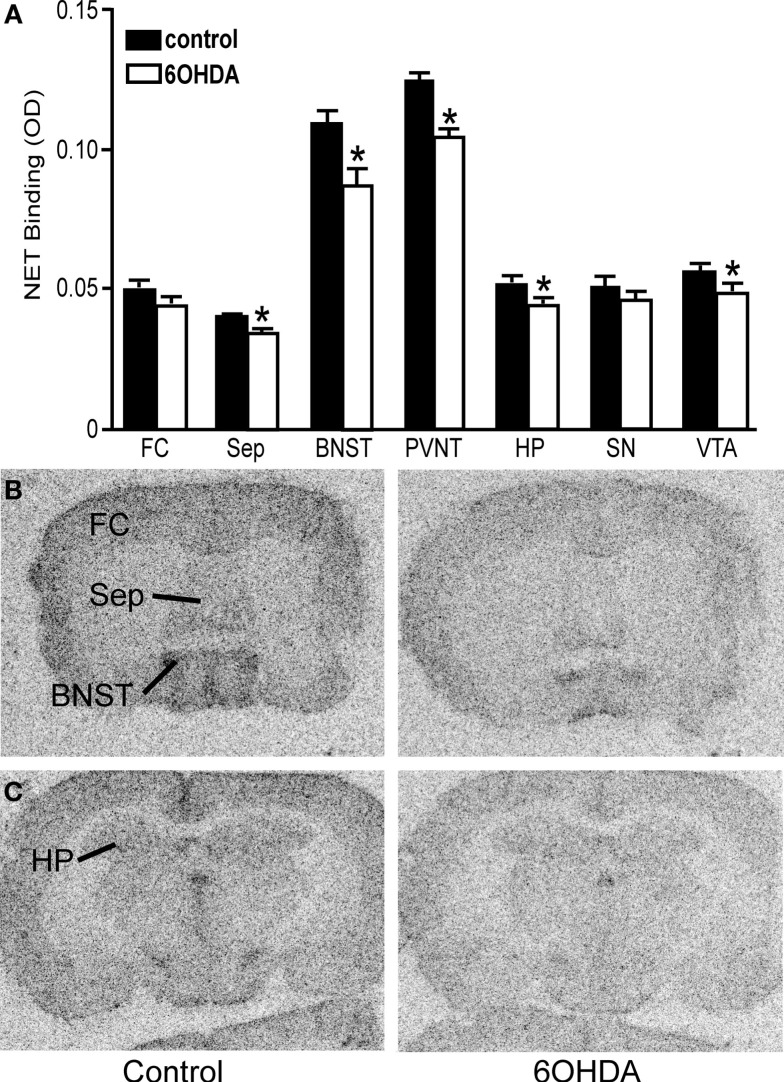
**Bilateral LC neuronal loss reduces NET binding sites in specific forebrain regions 3 days after bilateral vehicle/6OHDA administration**. **(A)** Quantification of NET binding sites (OD) in control and 6OHDA-treated animals in forebrain regions. *Indicates significant difference to vehicle. Photomicrographs of NET binding sites at the level of the septum **(B)** and HP **(C)** of vehicle or 6OHDA-treated animal.

**Figure 3:**
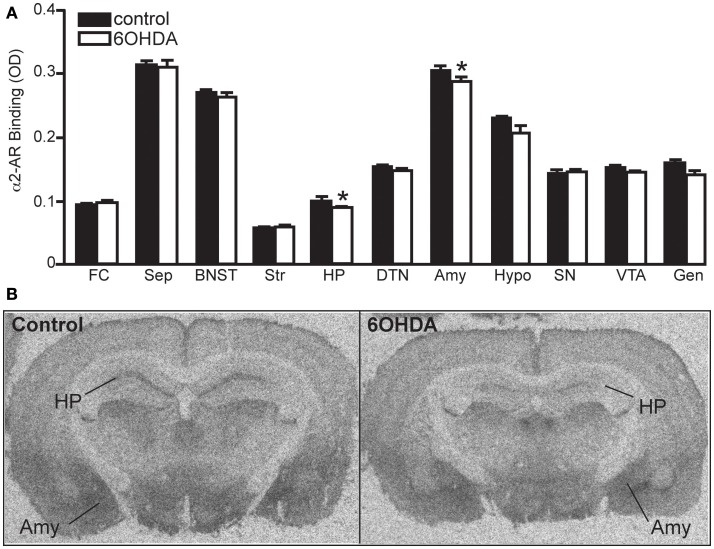
**Bilateral LC neuronal loss reduces α_2_-AR binding sites in the HP and Amy 3 days later**. **(A)** Quantification of α_2_-AR binding sites (OD) in control and 6OHDA-treated animals in forebrain regions and **(B)** photomicrographs of α_2_-AR binding in HP and Amy of control and 6OHDA-treated animal. *Indicates significant difference to vehicle.

**Figure 4:**
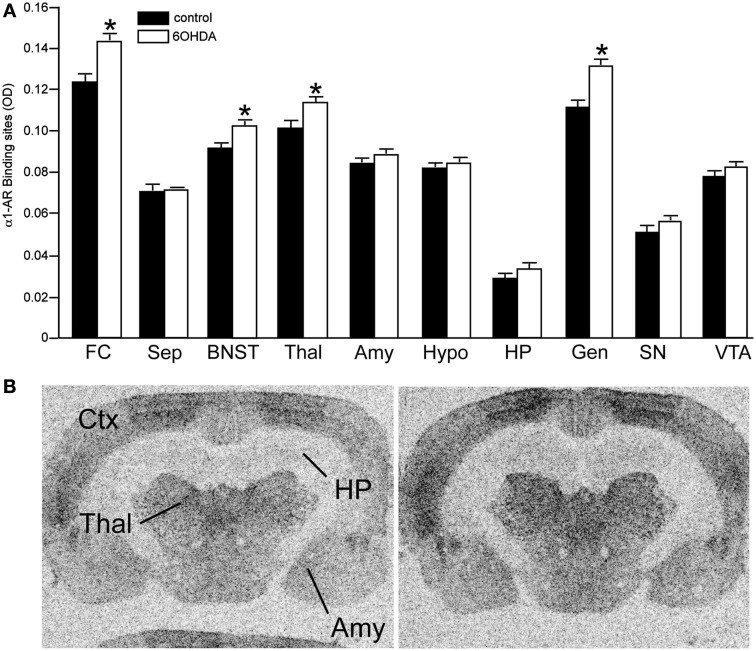
**Bilateral LC neuronal loss increases α_1_-AR binding sites in specific forebrain regions 3 days after bilateral vehicle/6OHDA administration**. **(A)** Quantification of α_1_-AR binding sites (OD) in control and 6OHDA-treated animals in forebrain regions and **(B)** photomicrographs of α_1_-AR binding in Ctx, Thal, HP, and Amy of control and 6OHDA-treated animals. *Indicates significant difference to vehicle.

Bilateral administration of 6OHDA directly into the LC significantly reduced NET binding sites in the sep, BNST, PVNT, HP, and VTA (Figure 2). Interestingly, the number of NET binding sites in the FC was not significantly different from that in control animals (13% reduced), but this region did demonstrate a significant reduction in NE concentration (31% reduced; Table 1). The reduction in NET binding sites observed 3 days after administration of 6OHDA directly into the LC was not as great as observed 3 weeks after 6OHDA (Szot et al., 2012). Bilateral administration of 6OHDA directly into the LC significantly reduced α_2_-AR only in the HP (11% reduced) and Amy (7% reduced; Figure 3). Three weeks after 6OHDA, α_2_-AR binding sites were reduced in the HP, but not in the Amy at this time (Szot et al., 2012). These data suggest that the damage to LC neurons 3 days after 6OHDA had not extended completely to the terminal regions in the forebrain. Interestingly, administration of 6OHDA significantly increased α_1_-AR binding sites in the FC, BNST, Thal, and Gen (Figure 4). This is in contrast to the effect of 6OHDA 3 weeks later where a reduction in α_1_-AR binding sites was observed in the Amy and SN (Szot et al., 2012).

### Experiment 2

#### Locus coeruleus

**Figure 5:**
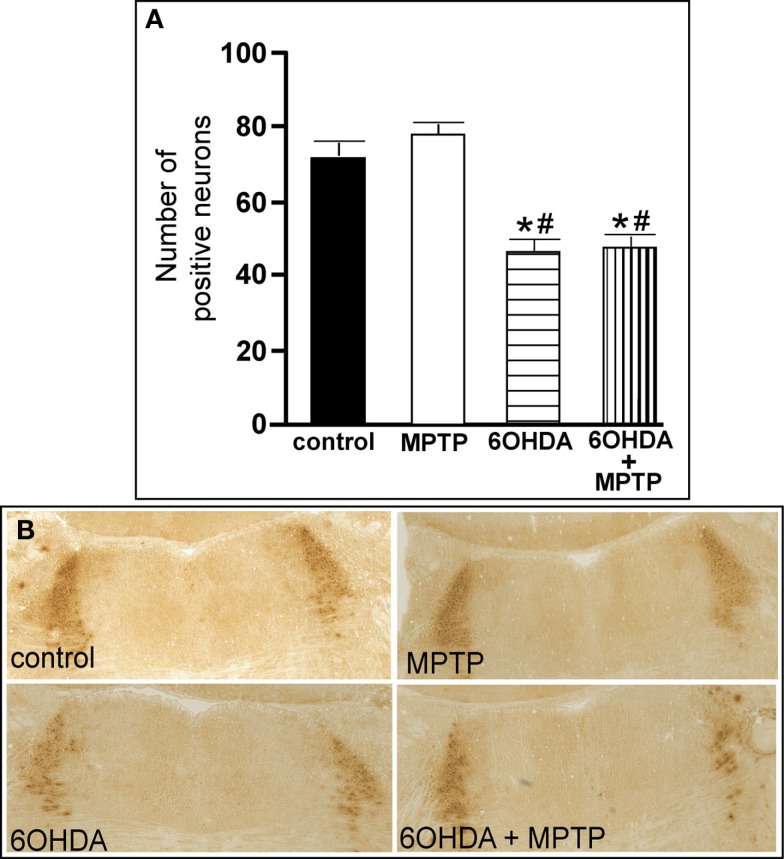
**Reduced number of TH-IR neurons in the LC of animals administered 6OHDA alone and with MPTP compared to control and MPTP-treated animals**. **(A)** Quantitation of the number of TH-IR neurons and **(B)** photomicrographs of TH-IR in the LC of animals which received saline, MPTP, 6OHDA, and 6OHDA + MPTP. *Indicates significant difference to control. ^#^Indicates significant difference to MPTP.

**Figure 6:**
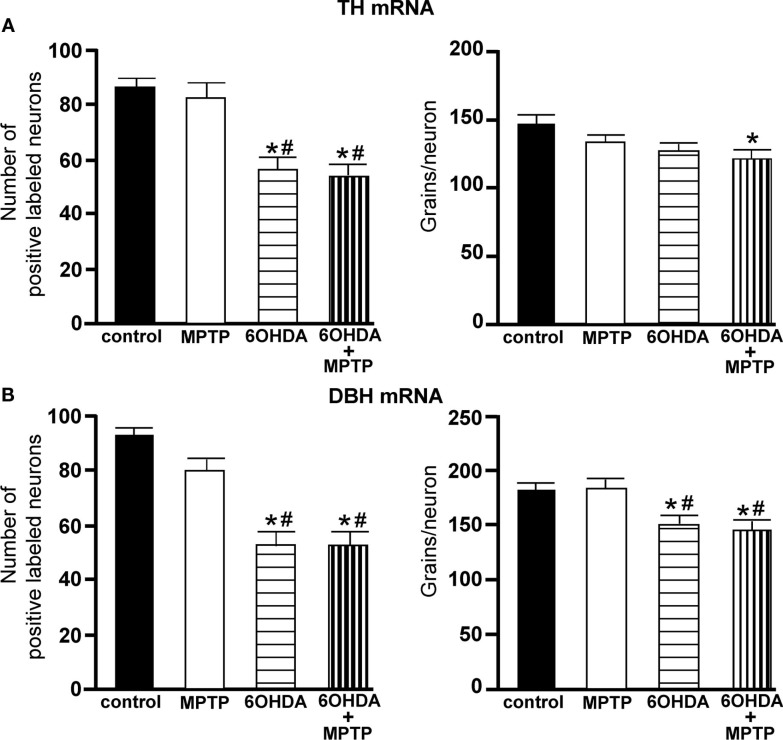
**Reduced number of TH and DBH positive labeled neurons and reduced expression/neuron in the LC of animals administered 6OHDA alone and with MPTP compared to control and MPTP-treated animals**. **(A)** Quantitation of the number of TH mRNA positive labeled neurons (left panel) and amount of TH mRNA expression/neuron (right panel) and **(B)** quantitation of the number of DBH mRNA positive labeled neurons (left panel) and amount of DBH mRNA expression/neuron (right panel) in the LC of animals which received saline, MPTP, 6OHDA, and 6OHDA + MPTP. *Indicates significant difference to control. ^#^Indicates significant difference to MPTP.

The number of TH-IR neurons in the LC of animals administered MPTP was not significantly different from control animals (Figure 5), indicating that MPTP does not affect the number of noradrenergic neurons in the LC. However, bilateral LC administration of 6OHDA resulted in a significant reduction in the number of TH-IR neurons in the LC as compared to control (36% reduced) and MPTP (41% reduced)-treated animals, and the addition of MPTP 3 days after 6OHDA did not further enhance the reduction induced by 6OHDA (34% reduced compared to control and 39% reduced compared to MPTP-treated animals; Figure 5). The effect of 6OHDA on LC neuronal number was variable; the range of neuronal loss due to 6OHDA with or without MPTP was ∼15–70%. The number of TH- and DBH mRNA positive labeled neurons in the LC of all four treatment groups is in agreement with data generated with TH-IR (Figures 6A,B left panels); MPTP had no effect on the number of TH- and DBH mRNA positive labeled neurons in the LC, and 6OHDA alone or in combination with MPTP significantly reduced the number of LC noradrenergic neurons compared to control or MPTP-treated animals (Figures 6A,B left panels).

The amount of TH mRNA expression/neuron in LC neurons was not affected by the addition of MPTP or 6OHDA alone, but the combination of 6OHDA + MPTP resulted in a significant reduction (17% reduced) in TH mRNA expression/neuron compared to control group (Figure 6A right panel). The lack of an effect of 6OHDA on LC TH mRNA expression/neuron was also observed 3 days after bilateral 6OHDA (Figure 1B). In contrast, the amount of DBH mRNA expression/neuron in 6OHDA-treated animals was significantly reduced 3 weeks later compared to control (17% reduced), and MPTP-treated (17% reduced) animals Figure 6B right panel); a response similar to 3 days after 6OHDA (Figure 1B). The reduction in DBH mRNA expression/neuron produced by 6OHDA is not enhanced with the administration of MPTP (20% reduction; Figure 6B right panel). MPTP did not affect DBH mRNA expression/neuron in the LC (Figure 6B right panel).

#### Substantia nigra

**Figure 7:**
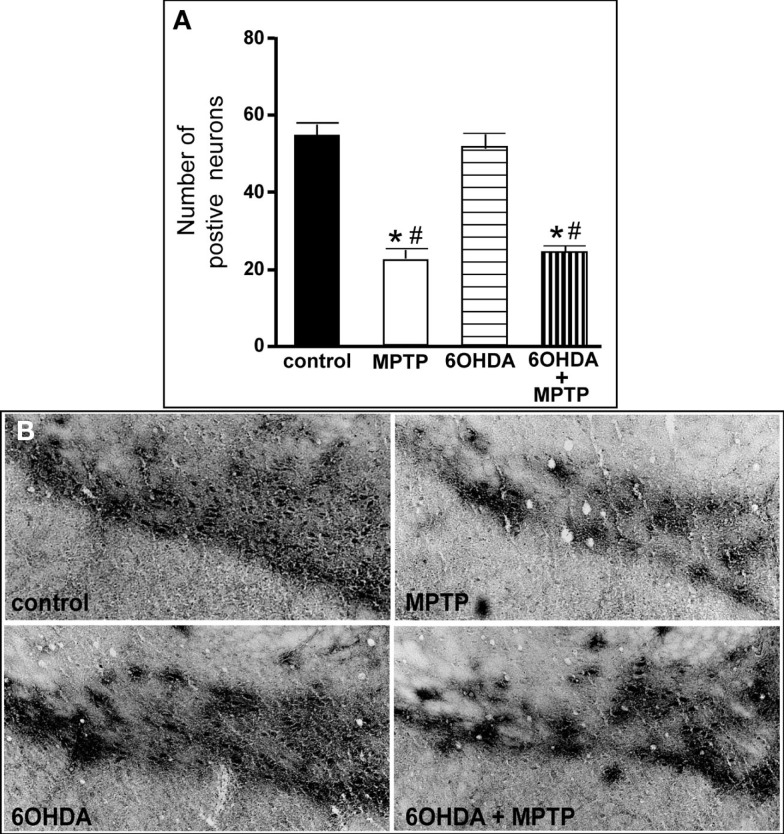
**Reduced number of TH-IR neurons in the SN of animals administered MPTP alone and with 6OHDA compared to control and 6OHD-treated animals**. **(A)** Quantitation of the number of TH-IR neurons and **(B)** photomicrographs of TH-IR in the SN of animals which received saline, MPTP, 6OHDA, and 6OHDA + MPTP. *Indicates significant difference to control. ^#^Indicates significant difference to 6OHDA.

**Figure 8:**
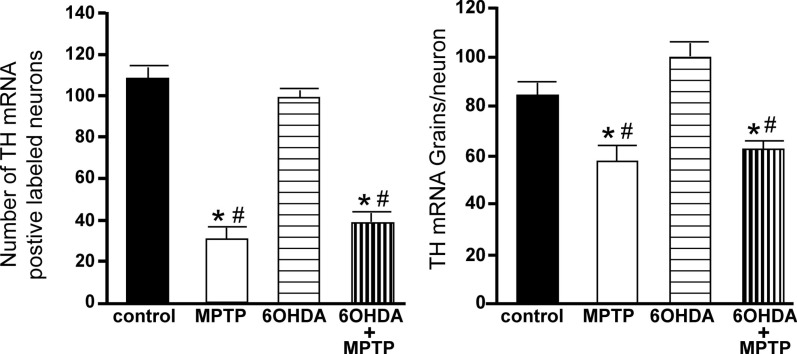
**Reduced number of TH positive labeled neurons and reduced TH mRNA expression/neuron in the SN of animals administered MPTP alone and with 6OHDA compared to control and 6OHDA-treated animals**. Quantitation of the number of TH mRNA positive labeled neurons (left panel) and amount of TH mRNA expression/neuron (right panel) in the SN of animals which received saline, MPTP, 6OHDA, and 6OHDA + MPTP. *Indicates significant difference to control. ^#^Indicates significant difference to 6OHDA.

The number of TH-IR neurons in the SN of animals administered 6OHDA was not significantly different from control animals (Figure 7), indicating that administration of 6OHDA directly into the LC did not affect the number of dopaminergic neurons in the SN. However, administration of MPTP resulted in a significant reduction in the number of SN TH-IR neurons as compared to control (59% reduced) and 6OHDA-treated animals (57% reduced), and the addition of MPTP 3 days after 6OHDA did not alter the reduction induced by MPTP as compared to control (56% reduced) and 6OHDA (53% reduced)-treated animals (Figure 7). The effect of MPTP on SN neuronal number was variable; the range of neuronal loss due to MPTP with or without 6OHDA was ∼30–80%. There was no significant correlation of LC TH-IR neuronal number with the number of TH-IR neurons in the SN within any group, indicating that LC noradrenergic neurons did not affect the number of dopaminergic SN neurons. The number of TH mRNA positive labeled neurons in the SN of all four treatment groups was in agreement with data generated with TH-IR (Figure 8); 6OHDA did not significantly affect the number of SN TH mRNA positive labeled neurons, while MPTP alone or in combination with 6OHDA significantly reduced the number of SN dopaminergic neurons compared to control treated animals (MPTP: 71% reduced; 6OHDA + MPTP: 64% reduced) or 6OHDA (MPTP: 69% reduced; 6OHDA + MPTP: 61% reduced; Figure 8). Prior administration of 6OHDA did not exacerbate MPTP-induced reduction of the number of TH mRNA positive labeled neurons in the SN (Figure 8).

The amount of TH mRNA expression/neuron in SN neurons was not affected by the bilateral LC administration of 6OHDA; however administration of MPTP alone or with 6OHDA resulted in a significant reduction in TH mRNA expression/neuron compared to control (MPTP: 31% reduced; 6OHDA + MPTP: 25% reduced) and 6OHDA-treated animals (MPTP: 42% reduced; 6OHDA + MPTP: 38% reduced; Figure 8). The prior administration of 6OHDA to MPTP did not exacerbate MPTP damage on SN neurons.

#### Ventral tegmental area

**Figure 9:**
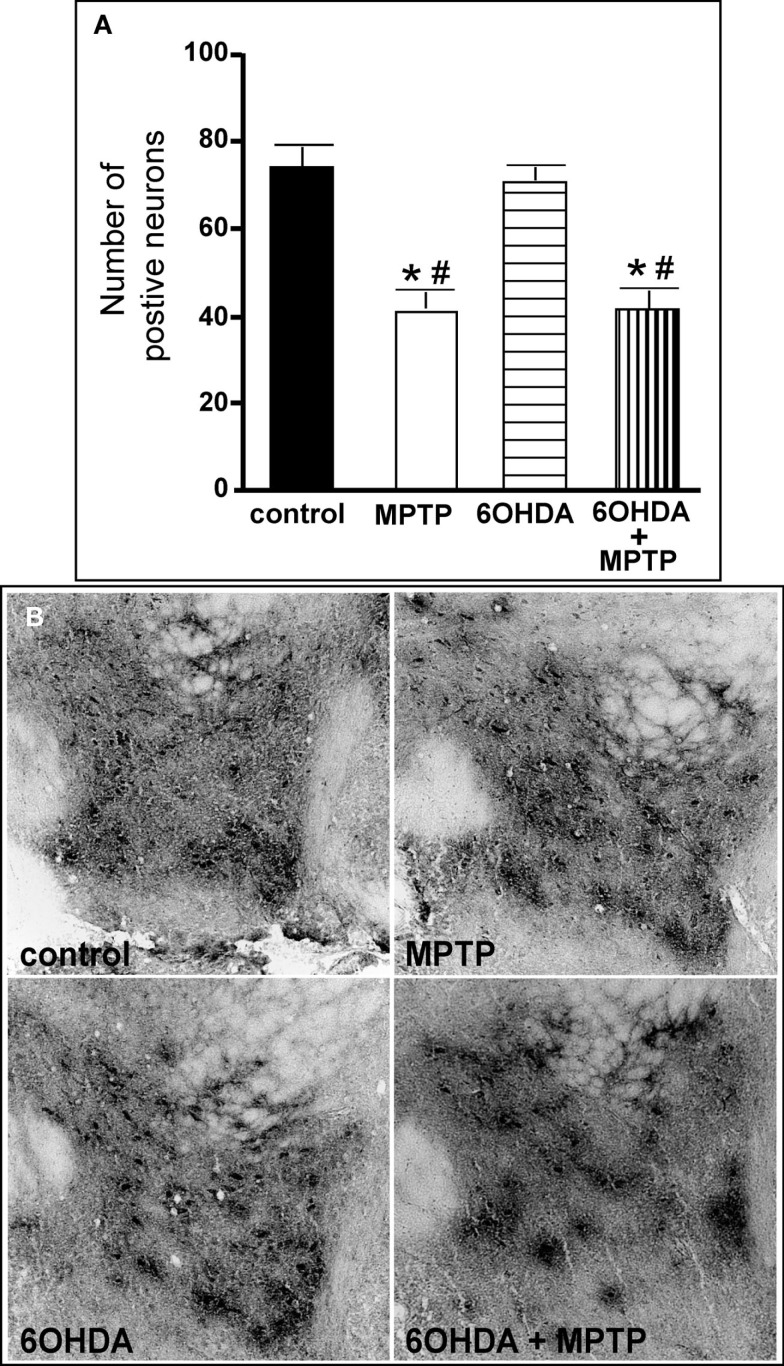
**Reduced number of TH-IR neurons in the VTA of animals administered MPTP alone and with 6OHDA compared to control and 6OHD-treated animals**. **(A)** Quantitation of the number of TH-IR neurons and **(B)** photomicrographs of TH-IR in the VTA of animals which received saline, MPTP, 6OHDA, and 6OHDA + MPTP. *Indicates significant difference to control. ^#^Indicates significant difference to 6OHDA.

**Figure 10:**
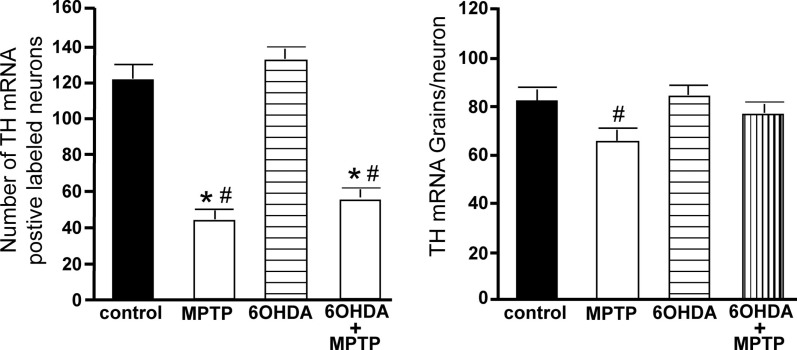
**Reduced number of TH positive labeled neurons and reduced TH mRNA expression/neuron in the VTA of animals administered MPTP alone and with 6OHDA compared to control and 6OHD-treated animals**. Quantitation of the number of TH mRNA positive labeled neurons (left panel) and amount of TH mRNA expression/neuron (right panel) in the VTA of animals which received saline, MPTP, 6OHDA, and 6OHDA + MPTP. *Indicates significant difference to control. ^#^Indicates significant difference to 6OHDA.

The number of TH-IR neurons in the VTA of animals administered 6OHDA was not significantly different from control animals (Figure 9), indicating that administration of 6OHDA directly into the LC did not affect the number of dopaminergic neurons in the VTA. However, administration of MPTP resulted in a significant reduction in the number of TH-IR neurons as compared to control (44% reduced) and 6OHDA-treated animals (42% reduced), and the addition of MPTP 3 days after 6OHDA did not alter the reduction induced by MPTP as compared to control (44% reduced) and 6OHDA-treated animals (41% reduced; Figure 9). The effect of MPTP on VTA neuronal number was variable; the range of neuronal loss due to MPTP with or without 6OHDA was ∼15–77%. There was no significant correlation of LC TH-IR neuronal number with the number of TH-IR neurons in the VTA within any group, indicating LC neuronal number did not affect dopaminergic neurons in the VTA. The number of TH mRNA positive labeled neurons in the VTA in all four groups was in agreement with data generated with TH-IR (Figure 10 left panel); 6OHDA had no effect on the number of TH mRNA positive labeled neurons, while MPTP alone or in combination with 6OHDA significantly reduced the number of VTA dopaminergic neurons compared to control (MPTP: 64% reduced; 6OHDA + MPTP: 56% reduced) or 6OHDA-treated animals (MPTP: 67% reduced; 6OHDA + MPTP: 59% reduced; Figure 10 left panel). Prior administration of 6OHDA did not exacerbate MPTP-induced reduction of the number of TH mRNA positive labeled neurons in the VTA (Figure 10 left panel).

The amount of TH mRNA expression/neuron in VTA neurons was not affected by bilateral LC administration of 6OHDA; however, TH mRNA expression/neuron in VTA neurons was significantly reduced in animals administered MPTP compared to 6OHDA-treated animals (23% reduced; Figure 10 right panel). The effect of MPTP on TH mRNA expression/neuron in VTA is reversed when 6OHDA is administered prior to MPTP (Figure 10 right panel).

#### Catecholamine concentrations in striatum

NE concentration in the striatum was not significantly affected by the administration of MPTP or 6OHDA or the combination of 6OHDA + MPTP, although there was a trend for a reduction with the combination of 6OHDA + MPTP (33% reduced). Interestingly, DHPG concentration (NE metabolite) was significantly reduced only in the 6OHDA + MPTP-treated animals compared to control animals (36% reduced; Table 2). DOPA concentration in the striatum was significantly reduced in all three treatment groups compared to control animals (58% reduced in both MPTP alone and 6OHDA + MPTP, and 33% reduced in 6OHDA, respectively). In addition DOPA concentration in 6OHDA + MPTP-treated animals was significantly reduced compared to 6OHDA-treated animals (Table 2). DA and its metabolite DOPAC, were also significantly reduced in all three treatment groups compared to the control group (MPTP: 91% reduced DA, 85% reduced DOPAC; 6OHDA: 29% reduced DA, 33% reduced DOPAC, and 6OHDA + MPTP: 91% reduced DA, 87% reduced DOPAC). MPTP- and 6OHDA + MPTP-treated animals were significantly reduced compared to 6OHDA-treated animals (Table 2). The reduced concentrations of DA and DOPAC concentration in the striatum resulting from the combined administration of 6OHDA + MPTP were not significantly different from the DA and DOPAC concentrations of MPTP-treated animals, reflecting the lack of an effect of LC neuronal loss on MPTP-induced damage on dopaminergic neurons in the SN and VTA. Interestingly, the administration of 6OHDA significantly reduced the concentration of DOPA, DA, and DOPAC but not NE and DHPG in the striatum indicating that a loss of noradrenergic neurons indirectly affects striatal DA concentration.

**Table 2:** **Catecholamine levels expressed as ng per catecholamine/mg protein in striatum of control, MPTP alone, 6OHDA alone, and 6OHDA + MPTP-treated animals**.

Group	DHPG	NE	DOPA	DA	DOPAC
Control	0.11 ± 0.008	1.62 ± 0.19	0.12 ± 0.02	122.3 ± 9.7	13.5 ± 1.3
MPTP	0.09 ± 0.009	1.52 ± 1.6	0.05 ± 0.006*	11.3 ± 2.7*^#^	2.0 ± 0.4 *^#^
60HDA	0.09 ± 0.008	1.48 ± 0.15	0.08 ± 0.007*	87.0 ± 5.2*	9.0 ± 0.74*
60HDA + MPTP	0.07 ± 0.007*	1.09 ± 0.12	0.05 ± 0.003*^#^	10.9 ± 1.5*^#^	1.8 ± 0.23*^#^

*Indicates significant difference from control animals.

*^#^Indicates significant difference from 60HDA alone animals*.

#### Correlation of LC, SN, and VTA neuronal number to striatal catecholamine concentration

The number of LC noradrenergic neurons in the control group was not significantly correlated with any catecholamine concentration in the striatum (Table 3). In contrast, the number of LC noradrenergic neurons in the MPTP, 6OHDA, and 6OHDA + MPTP-treated groups were significantly correlated with NE and DHPG in the striatum (Table 3), indicating that when the noradrenergic system was compromised, the loss of LC neurons reduces NE and DHPG concentrations in the striatum. The number of SN dopaminergic neurons in the control group was not significantly correlated with any catecholamine concentration in the striatum (Table 3). The number of SN dopaminergic neurons in the MPTP-treated group correlated only with striatal DHPG and NE, indicating a loss of dopaminergic neurons due to MPTP can influence striatal NE and DHPG concentrations, but not DA, DOPA, and DOPAC (Table 3). Only when the combination of 6OHDA + MPTP was administered was there a significant correlation of the number of SN dopaminergic neurons with striatal DOPA, DA, and DOPAC, but there was no significant correlation with NE or DHPG (Table 3). The VTA region showed exactly the same correlations as the SN. The number of VTA dopaminergic neurons in the control group was not significantly correlated with any catecholamine concentration in the striatum (Table 3). The number of VTA dopaminergic neurons in the MPTP group correlated to striatal DHPG and NE; while the number of VTA dopaminergic neurons in the 6OHDA + MPTP group correlated with striatal DOPA, DA, and DOPAC. It is interesting to note that administration of MPTP resulted in a significant correlation of the number of dopaminergic neurons with the concentrations of NE and its metabolite in the striatum, but not with DOPA, DA, or its metabolite DOPAC. Only the combined administration of 6OHDA + MPTP resulted in a significant correlation of dopaminergic neurons in the SN and VTA with striatal concentrations of DOPA, DA, and DOPAC.

**Table 3:** **Correlation between the number of TH-IR neurons in the LC, SN, and VTA to striatal catecholamine concentration**.

Region/treatment	DHPG	NE	DOPA	DA	DOPAC
LC #/Control	*P* = 0.62	*P* = 0.84	*P* = 0.21	*P* = 0.94	*P* = 0.14
	*r^2^* = 0.018	*r*^2^ = 0.003	*r^2^* = 0.11	*r^2^* = 0.0005	*r*^2^ = 0.15
LC #/MPTP	*P* = 0.047*	*P* = 0.037*	*P* = 0.56	*P* = 0.99	*P* = 0.81
	*r*^2^ = 0.25	*r*^2^ = 0.28	*r*^2^ = 0.03	*r*^2^ = 5 × 10^−6^	*r*^2^ = 0.004
LC #/60HDA	*p* = 0.001*	*P* = 0.0008*	*P* = 0.23	*P* = 0.11	*P* = 0.24
	*r*^2^ = 0.42	r* = 0.44	*r*^2^ = 0.06	*r*^2^ = 0.12	*r*^2^ = 0.07
LC #/60HDA + MPTP	*P* = 0.002*	*P* = 0.002*	*P* = 0.49	*P* = 0.77	*P* = 0.71
	*r*^2^ = 0.48	*r*^2^ = 0.35	*r*^2^ = 0.02	*r*^2^ = 0.004	*r*^2^ = 0.006
SN #/Control	*P* = 0.90	*P* = 0.92	*P* = 0.54	*P* = 0.97	*P* = 0.74
	*r*^2^ = 0.001	*r^2^ *= 0.0008	*r*^2^ = 0.03	*r*^2^ = 0.0001	*r*^2^ = 0.003
SN #/MPTP	*p* = 0.01*	*P* = 0.042*	*P* = 0.76	*P* = 0.28	*P* = 0.09
	*r*^2^ = 0.39	*r*^2^ = 0.26	*r*^2^ = 0.007	*r*^2^ = 0.08	*r*^2^ = 0.19
SN #/60HDA	*P* = 0.47	*P* = 0.23	*P* = 0.45	*P* = 0.76	*P* = 0.94
	*r*^2^ = 0.03	*r*^2^ = 0.08	*r*^2^ = 0.03	*r*^2^ = 0.005	*r*^2^ = 0.0003
SN #60HDA + MPTP	*P* = 0.97	*P* = 0.92	*P* = 0.002*	*P* = 0.0009*	*p* = 0.001*
	*r*^2^ = 6 × 10^−5^	*r*^2^ = 0.0004	*r*^2^ = 0.36	*r*^2^ = 0.41	*r*^2^ = 0.39
VTA #/Control	*P* = 0.54	*P* = 0.65	*P* = 0.09	*P* = 0.69	*P* = 0.43
	*r*^2^ = 0.03	*r*^2^ = 0.02	*r*^2^ = 0.19	*r*^2^ = 0.012	*r*^2^ = 0.06
VTA #/MPTP	*P* = 0.024*	*P* = 0.022*	*P* = 0.35	*P* = 0.09	*P* = 0.06
	*r*^2^ = 0.31	*r*^2^ = 0.32	*r*^2^ = 0.06	*r*^2^ = 0.19	*r*^2^ = 0.24
VTA #/60HDA	*P* = 0.18	*P* = 0.71	*P* = 0.55	*P* = 0.69	*P* = 0.79
	*r*^2^ = 0.10	*r*^2^ = 0.008	*r*^2^ = 0.02	*r*^2^ = 0.009	*r*^2^ = 0.004
VTA #/60HDA + MPTP	*P* = 0.52	*P* = 0.21	*P* = 0.017*	*P* = 0.0002*	*P* = 0.0012*
	*r*^2^ = 0.02	*r*^2^ = 0.07	*r*^2^ = 0.23	*r*^2^ = 0.48	*r*^2^ = 0.39

## Discussion

### Three days after 6OHDA there is a significant loss of LC neurons and terminals

The focus of this study is to determine if the loss of LC noradrenergic neurons can enhance the damage of a dopaminergic neurotoxin on dopaminergic neurons. To assess if LC loss can enhance damage to dopaminergic neurons, MPTP is administered 3 days after bilateral administration of 6OHDA directly into the LC. At the time MPTP is administered, the effects of 6OHDA are restricted to LC neurons, no effect on noradrenergic lateral tegmental neurons and dopaminergic neurons is observed; results similar to data generated 3 weeks after 6OHDA (Figures 7–10; Szot et al., 2012). Three days after bilateral 6OHDA there is a significant reduction in: (1) the number of LC noradrenergic neurons as observed by TH-IR, TH-, and DBH mRNA expression; (2) DBH mRNA expression/neuron; (3) DHPG and NE concentration in the FC, HP, and Amy, with no effect on DOPA, DA, and DOPAC; and (4) NET and α_2_-AR binding sites in forebrain regions; results again similar to what is observed 3 weeks after 6OHDA (Figure 5; Tables 1 and 2; Szot et al., 2012). However, the reduction in many of these noradrenergic markers in forebrain regions 3 days after bilateral 6OHDA is less than that observed 3 weeks after unilateral 6OHDA (Szot et al., 2012), suggesting that 3 days after 6OHDA the LC terminals are not affected to the same degree as the loss of LC neurons. This hypothesis is supported by a lack of a significant correlation 3 days after 6OHDA of LC neuronal number with NE concentrations in the FC, HP, and Amy. Interestingly, 3 days after 6OHDA α_1_-AR binding sites are significantly elevated in the FC, BNST, Thal, and Gen; while 3 weeks after unilateral 6OHDA there is a significant loss of α_1_-AR in the Amy and SN. Though NE concentrations are not measured in the SN/VTA region 3 days after bilateral 6OHDA administration, data from 3 weeks after unilateral 6OHDA suggests that this region 3 days after 6OHDA may also have reduced NE concentration (Szot et al., 2010, 2012). Therefore when MPTP is administered 3 days after 6OHDA, there is a significant loss of LC neurons (60% reduced) and innervation to forebrain regions is compromised.

### Administration of MPTP affects noradrenergic neurons and 6OHDA affects dopaminergic neurons

Administration of MPTP significantly reduces the number of SN and VTA dopaminergic neurons (greater effect on SN neurons than VTA neurons) and decreases TH mRNA expression/neuron in dopaminergic neurons of the SN. MPTP also reduces striatal DOPA, DA, and DOPAC concentrations, supporting the selectivity of MPTP to dopaminergic neurons. These changes in the dopaminergic nervous system have been reported numerous times in mice and these changes have been observed in dopaminergic neurons of postmortem PD subjects (Heikkila et al., 1984; Javoy-Agid et al., 1990; Kastner et al., 1993; Jackson-Lewis et al., 1995; Jakowec et al., 2004; Nagatsu and Sawada, 2007). Interestingly, the number of SN or VTA neurons following MPTP treatment does not correlate with DA, DOPA, or DOPAC concentrations in the striatum (Table 3), suggesting some other factor may be influencing the dopaminergic neurons and/or DA concentration in the striatum. Previous work has demonstrated that MPTP in mice has a more dramatic effect on striatal DA levels than on dopaminergic neurons because it has been hypothesized that MPTP initially affects dopaminergic terminals and with time the effect of MPTP on terminals may be reversed (Heikkila et al., 1984; Perry et al., 1985; Donnan et al., 1986; Ricaurte et al., 1986; Jackson-Lewis et al., 1995; Bezard et al., 2000; Jakowec et al., 2004). This effect of MPTP at dopaminergic terminals may explain the lack of a correlation between SN and VTA neuronal number to striatal DOPA, DA, and DOPAC concentrations.

MPTP does not significantly affect the number of LC noradrenergic neurons as measured by TH-IR, and TH and DBH mRNA expression, and it does not affect the expression/neuron of TH and DBH mRNA in LC neurons. Consequently, there is not a significant effect of MPTP on striatal DHPG and NE concentrations. Interestingly though, in the MPTP-treated animals NE and DHPG concentrations in the striatum are significantly correlated with SN and VTA neurons. The reduction in DOPA concentration due to MPTP could result in reduced NE concentration in the striatum because DOPA is also a precursor for NE, but as described above, MPTP-induced loss of SN and VTA neurons does not correlate with DOPA concentration in the striatum. Therefore, the correlation of SN and VTA dopaminergic neurons following MPTP treatment could not result in reduced striatal NE and DHPG levels. Dopaminergic neurons in the SN and VTA have been shown to innervate the LC (MacRae-Degueurce and Milon, 1983); therefore, the effect of MPTP on NE and DHPG concentrations in the striatum may be due to lower basal firing rate of LC neurons when dopaminergic neuronal numbers are reduced (Miguelez et al., 2011).

The loss of LC noradrenergic neurons does not affect the number of dopaminergic neurons in the SN or VTA as described above or in previously published work (Szot et al., 2012), but the loss of LC noradrenergic neurons does reduce DA, DOPA, and DOPAC concentrations in the striatum, which also has been shown previously (Lategan et al., 1990, 1992). LC neurons innervate dopaminergic neurons in the SN and VTA (Swanson and Hartman, 1975; Jones and Moore, 1977; Phillipson, 1979; Simon et al., 1979; Jones and Yang, 1985; Fritschy and Grzanna, 1990; Szot et al., 2012); and a reduction in LC neurons reduces the activity of dopaminergic neurons (Grenhoff and Svensson, 1989, 1993; Grenhoff et al., 1993, 1995; Guiard et al., 2008; Wang et al., 2010). This effect is mediated by dopamine D2 receptors, not α_1_- or α_2_-ARs (Arencibia-Albite et al., 2007). The reduced activity of dopaminergic neurons as a result of LC neuronal loss and innervation could result in reduced DA, DOPA, and DOPAC concentration in the striatum.

### Combination of 6OHDA + MPTP does not enhance SN and VTA neuronal loss or striatal DA concentration, but it does result in a correlation of SN and VTA neurons to striatal DOPA, DA, and DOPAC concentration

The administration of MPTP after 6OHDA did not affect the loss of LC noradrenergic neurons compared to 6OHDA, but the combination of 6OHDA + MPTP did result in a significant reduction of TH mRNA expression/neuron in surviving LC neurons and striatal DHPG concentration (NE metabolite) compared to control animals. The reduction in TH mRNA expression/neuron in 6OHDA + MPTP animals may be the reason for the reduction in striatal DHPG concentration. NE concentration in the striatum of 6OHDA + MPTP is reduced compared to control animals (33% reduced), but it did not reach statistical significance. The number of LC noradrenergic neurons in the 6OHDA + MPTP group was highly correlated with striatal NE and DHPG concentration, similar to 6OHDA-treated group.

In contrast to previously published work (Mavridis et al., 1991; Marien et al., 1993; Bing et al., 1994; Fornai et al., 1996, 1997; Srinivasan and Schmidt, 2003, 2004; Archer and Fredriksson, 2006) the dosing paradigm of 6OHDA and MPTP outlined in this study did not enhance the loss of dopaminergic neurons in the SN and VTA or further reduce the concentration of DOPA, DA, or DOPAC in the striatum, despite the striatal reduction of DA, DOPA, and DOPAC by 6OHDA alone. This is the only study to date that measures neuronal numbers in the LC, SN, and VTA in conjunction with striatal catecholamine levels. The lack of effect of LC loss on MPTP-induced dopaminergic damage may be that the reduction of NE concentration at the time MPTP was administered is not severe enough, 3 days following 6OHDA NE loss in forebrain regions is moderate (20% reduced in HP, 30% reduced in FC, and 33% in Amy). The 3-day time was chosen for this study because it falls in the middle of the times used in these previous published studies (same day – 3 weeks) where enhanced damage to dopaminergic neurons was observed following the administration of a noradrenergic neurotoxin (Mavridis et al., 1991; Marien et al., 1993; Bing et al., 1994; Fornai et al., 1995, 1996, 1997; Srinivasan and Schmidt, 2003, 2004; Archer and Fredriksson, 2006). It is difficult to know exactly how severe a loss of NE concentration was in forebrain regions of these published studies when the dopaminergic neurotoxin was administered because the majority of these studies did not assess alterations in the noradrenergic system. However, the enhanced effect of a noradrenergic neurotoxin on dopaminergic damage by a dopaminergic neurotoxin can not be due solely to reduced forebrain NE concentration because in two of the studies, MPTP was administered too close in time (same time or 24 h later) to the noradrenergic neurotoxin (6OHDA in LC and DSP4; Mavridis et al., 1991; Marien et al., 1993) for their to be reduced NE concentration in the forebrain. In addition, administration of MPTP to the *Dbh* knockout mice, which are unable to synthesize NE (Thomas et al., 1998), does not enhance MPTP-induced damage (Rommelfanger and Weinshenker, 2007); therefore, concentration of NE in forebrain regions does not appear to be an influence in mediating the enhanced damage induced by MPTP. Loss of LC noradrenergic neurons and innervation may be an influencing factor in mediating the enhanced damage of dopaminergic neurotoxins; however this study indicates that the reduced number of LC noradrenergic neurons after 6OHDA + MPTP does not correlate with the reduced number of SN or VTA neurons, indicating the degree of LC neuronal loss does not influence the number of dopaminergic neurons. Previous work demonstrating enhanced dopaminergic damage did not assess LC neuronal loss to dopaminergic neurons after dopaminergic neurotoxin.

Interestingly, the combination of 6OHDA + MPTP did result in a significant correlation between the number of SN and VTA neurons to striatal DOPA, DA, and DOPAC concentration, a response not observed with MPTP alone. The significant correlation of SN and VTA neuronal number after MPTP to striatal NE and DHPG concentration is lost when 6OHDA is administered prior to the administration of MPTP. It is unclear why the combination of LC neuronal loss before dopaminergic neuronal loss results in a correlation between dopaminergic neuronal numbers to striatal DOPA, DA, and DOPAC concentrations. Even though MPTP significantly reduces striatal DA concentration, mice do not exhibit motor deficits that are observed in humans. When noradrenergic neurons are reduced in monkey models of PD, the motor deficits observed with MPTP are more severe and persistent (Mavridis et al., 1991; Alexander et al., 1992). These data indicate that the loss of LC noradrenergic neurons is an important aspect of PD. Rommelfanger et al. (2007) showed that *Dbh* knockout mice were profoundly impaired on most motor tests, but an 80% loss of striatal DA in mice does not result in motor deficits.

### Summary

The loss of LC neurons and forebrain innervation prior to the administration of MPTP does not result in an enhanced loss of dopaminergic neurons in the SN and VTA or DA concentration in the striatum. However, when 6OHDA and MPTP are sequentially administered to animals a correlation now exists between SN and VTA neuronal number with striatal DA. This may occur because administration of 6OHDA or MPTP alone can affect the function of the other region’s neurons. 6OHDA induced LC neuronal loss results in reduced DOPA, DA, and DOPAC in the striatum but does not affect NE and DHPG. MPTP alone does not significantly reduce NE or DHPG, but the combination of 6OHDA + MPTP significantly reduces LC TH mRNA expression/neuron and striatal DHPG concentration. In PD, the interaction between noradrenergic and dopaminergic regions is altered due to neuronal loss in both regions, and this altered interaction may influence the efficacy of pharmacological treatment of PD. Recently, Barnum et al. (2012) demonstrated a difference in the response of rats to L-DOPA therapy between animals that had only a dopaminergic lesion to animals with a noradrenergic and dopaminergic lesion. These results indicate that the loss of LC and SN/VTA neurons in PD affect the function of the surviving neurons in the alternate region; therefore, exploring this interaction will aid in understanding PD.

## Conflict of Interest Statement

The authors declare that the research was conducted in the absence of any commercial or financial relationships that could be construed as a potential conflict of interest.

## References

[B1] Alexander G. M. Schwartzman R. J. Brainard L. Gordon S. W. Grothusen J. R. (1992). Changes in brain catecholamines and dopamine uptake sites at different stages of MPTP parkinsonism in monkeys. Brain Res. 588, 261–269 10.1016/0006-8993(92)91584-2 1356591

[B2] Antelman J. E. Caggiula A. R. (1977). Norepinephrine-dopamine interaction and behavior. Science 195, 646–653 10.1126/science.841304 841304

[B3] Archer T. Fredriksson A. (2006). Influence of noradrenaline denervation on MPTP-induced deficits in mice. J. Neural Transm. 113, 1119–1129 10.1007/s00702-005-0402-5 16362627

[B4] Arencibia-Albite F. Paladini C. Williams J. T. Jimenez-Rivera C. A. (2007). Noradrenergic modulation of the hyperpolarization-activated cation current (Ih) in dopamine neurons of the ventral tegmental area. Neuroscience 149, 303–314 10.1016/j.neuroscience.2007.08.009 17884297 PMC2254936

[B5] Barnum C. J. Bhide N. Lindenbach D. Surrena M. A. Goldenberg A. A. Tignor S. (2012). Effects of noradrenergic denervation on L-DOPA-induced dyskinesia and its treatment by α- and β-adrenergic receptor antagonists in hemiparkinsonian rats. Pharmacol. Biochem. Behav. 100, 607–615 10.1016/j.pbb.2011.09.009 21978941 PMC3242909

[B6] Bertrand E. Lechowicz W. Szpak G. M. Dynecki J. (1997). Qualitative and quantitative analysis of locus coeruleus neurons in Parkinson’s disease. Folia Neuropathol. 35, 80–86 9377080

[B7] Bezard E. Dovero S. Imbert C. Boraud T. Gross C. E. (2000). Spontaneous long-term compensatory dopaminergic sprouting in MPTP-treated mice. Synapse 38, 363–368 10.1002/1098-2396(20001201)38:3<363::AID-SYN16>3.3.CO;2-1 11020240

[B8] Bing G. Zhang Y. Watanabe Y. McEwen B. S. Stone E. A. (1994). Locus coeruleus lesions potentiate neurotoxic effects of MPTP in dopaminergic neurons of the substantia nigra. Brain Res. 668, 261–265 10.1016/0006-8993(94)90534-7 7704612

[B9] Booze R. M. Hall J. A. Cress N. M. Miller G. D. Davis J. N. (1988). DSP-4 treatment produces abnormal tyrosine hydroxylase immunoreactive fibers in rat hippocampus. Exp. Neurol. 101, 75–86 10.1016/0014-4886(88)90066-0 2899031

[B10] Braak H. Rub U. Gai W. P. Tredici K. D. (2006). Cognitive decline correlates with neuropathological stage in Parkinson’s disease. J. Neurol. Sci. 248, 255–258 10.1016/j.jns.2006.05.011 16814807

[B11] Braak H. Tredici K. D. Rub U. de Vos R. A. I. Jansen Steur E. N. H. Braak A. (2003). Staging of brain pathology related to sporadic Parkinson’s disease. Neurobiol. Aging 24, 197–211 10.1016/S0197-4580(02)00065-9 12498954

[B12] Cash R. Dennis T. L’Heureux R. Raisman R. Javoy-Agid F. Scatton B. (1987). Parkinson’s disease and dementia: norepinephrine and dopamine in locus coeruleus. Neurology 37, 42–46 10.1212/WNL.37.1.42 3796837

[B13] Chan-Palay V. Asan E. (1989). Alterations in catecholamine neurons of the locus coeruleus in senile dementia of the Alzheimer’s type and in Parkinson’s disease with and without dementia and depression. J. Comp. Neurol. 287, 373–392 10.1002/cne.902870307 2570794

[B14] Chopin P. Colpaert F. C. Marien M. (1999). Effects of alpha-2-adrenoreceptor agonists and antagonists on circling behavior in rats with unilateral 6-hydroxydopamine lesions of the nigrostriatal pathway. J. Pharmacol. Exp. Ther. 288, 798–804 9918591

[B15] Damier P. Hirsch E. C. Agrid Y. Graybiel A. M. (1999). The substantia nigra of the human brain. II. Patterns of loss of dopaminergic neurons in Parkinson’s disease. Brain 122, 11437–11448 10.1093/brain/122.8.1421 10430830

[B16] Donnan G. A. Kaczmarczyk S. J. Solopotias T. Rowe P. Kalnins R. M. Vajda F. J. (1986). The neurochemical and clinical effects of 1-methyl-4-phenyl-1,2,3,6-tetrahydropyridine in small animals. Clin. Exp. Neurol. 22, 155–164 3495376

[B17] Fornai F. Alessandri M. G. Torracca M. T. Bassi L. Corsini G. U. (1997). Effects of noradrenergic lesions on MPTP/MPP+ kinetics and MPTP-induced nigrostriatal dopamine depletions. J. Pharmacol. Exp. Ther. 283, 100–107 9336313

[B18] Fornai F. Bassi L. Torracca M. T. Scalori V. Corsini G. U. (1995). Norepinephrine loss exacerbates methamphetamine-induced striatal dopamine depletion in mice. Eur. J. Pharmacol. 283, 99–102 10.1016/0014-2999(95)00313-A 7498327

[B19] Fornai F. Torracca M. T. Bassi L. D’Errigo D. A. Scalori V. Corsini G. U. (1996). Norepinephrine loss selectively enhances chronic nigrostriatal dopamine depletion in mice and rats. Brain Res. 735, 349–353 10.1016/0006-8993(96)00891-8 8911678

[B20] Fritschy J. M. Grzanna R. (1990). Distribution of locus coeruleus axons within the rat brainstem demonstrated by Phaseolus vulgaris leucoagglutinin anterograde tracing in combination with dopamine-β-hydroxylase immunofluorescence. J. Comp. Neurol. 293, 616–631 10.1002/cne.902930407 2329197

[B21] Gibb W. R. G. (1991). Neuropathology of the substantia nigra. Eur. Neurol. 31(Suppl.), 48–59 10.1159/000116721 1830274

[B22] Gibb W. R. G. Lees A. J. (1991). Anatomy, pigmentation, ventral and dorsal subpopulations of the substantia nigra, and differential cell death in Parkinson’s disease. J. Neurol. Neurosurg. Psychiatr. 54, 388–396 10.1136/jnnp.54.5.388 1865199 PMC488535

[B23] Goldstein D. S. Feuerstein G. Izzo J. L. Jr. Kopin I. J. Keiser H. R. (1981). Validity and reliability of liquid chromatography with electrochemical detection for measuring plasma levels of norepinephrine and epinephrine in man. Life Sci. 28, 467–475 10.1016/0024-3205(81)90139-9 7207028

[B24] Grenhoff J. Nissel M. Aston-Jones G. Svensson T. H. (1993). Noradrenergic modulation of midbrain dopamine cell firing elicited by stimulation of the locus coeruleus in the rat. J. Neural Transm. 93, 11–25 10.1007/BF01244934 8373553

[B25] Grenhoff J. North R. A. Johnson S. W. (1995). Alpha1-adrenrgic effects on dopamine neurons recorded intracellularly in the rat midbrain slice. Eur. J. Neurosci. 7, 1707–1713 10.1111/j.1460-9568.1995.tb00692.x 7582125

[B26] Grenhoff J. Svensson T. H. (1989). Clonidine modulates dopamine cell firing in rat ventral tegmental area. Eur. J. Pharmacol. 165, 11–18 10.1016/0014-2999(89)90765-6 2569981

[B27] Grenhoff J. Svensson T. H. (1993). Prazosin modulates the firing pattern of dopamine neurons in rat ventral tegmental area. Eur. J. Pharmacol. 233, 79–84 10.1016/0014-2999(93)90351-H 8097162

[B28] Grima B. Lamouroux A. Blanot F. Biguet N. F. Mallet J. (1985). Complete coding sequence of rat tyrosine hydroxylase mRNA. Proc. Natl. Acad. Sci. U.S.A. 82, 617–621 10.1073/pnas.82.2.617 2857492 PMC397092

[B29] Grimbergen Y. A. M. Langston J. W. Roos R. A. C. Bloem B. (2009). Postural instability in Parkinson’s disease: the adrenergic hypothesis and locus coeruleus. Expert Rev. Neurother. 9, 279–290 10.1586/14737175.9.2.279 19210201

[B30] Grzanna R. Berger U. Fritschy J.-M. Geffard M. (1989). Acute action of DSP-4 on central norepinephrine axons: biochemical and immunohistochemical evidence for differential effects. J. Histochem. Cytochem. 37, 1435–1442 10.1177/37.9.2768812 2768812

[B31] Guiard B. P. Mansari M. E. Merali Z. Blier P. (2008). Functional interactions between dopamine, serotonin and norepinephrine neurons: an in-vivo electrophysiological study in rats with monoaminergic lesions. Int. J. Neuropsychopharmacol. 11, 625–639 10.1017/S1461145707008383 18205979

[B32] Heikkila R. E. Hess A. Duvoisin R. C. (1984). Dopaminergic neurotoxicity of 1-methyl-4-phenyl-1,2,5,6-tetrahydropyridine in mice. Science 224, 1451–1453 10.1126/science.6610213 6610213

[B33] Hornykiewicz O. Kish S. J. (1987). Biochemical pathophysiology of Parkinson’s disease. Adv. Neurol. 45, 19–34 2881444

[B34] Hughes Z. A. Stanford S. C. (1998). A partial noradrenergic lesion induced by DSP4 increases extracellular noradrenaline concentration in rat frontal cortex: a microdialysis study in vivo. Psychopharmacology (Berl.) 136, 299–303 10.1007/s002130050569 9566816

[B35] Jackson-Lewis V. Jakowec M. Burke R. E. Przedborski S. (1995). Time course and morphology of dopaminergic neuronal death caused by the neurotoxin 1-methyl-4-phenyl-1,2,3,6-tetrahydropyridine. Neurodegeneration 4, 257–269 10.1016/1055-8330(95)90015-2 8581558

[B36] Jakowec M. W. Nixon K. Hogg E. McNeill T. Petzinger G. M. (2004). Tyrosine hydroxylase and dopamine transporter expression and following 1-methyl-4-pheyl-1,2,3,6-tetrahydropyridine-induced neurodegeneration of the mouse nigrostriatal pathway. J. Neurosci. Res. 76, 539–550 10.1002/jnr.20114 15114626

[B37] Javoy-Agid F. Hirsch E. C. Dumas S. Duyckaerts C. Mallet J. Agid Y. (1990). Decreased tyrosine hydroxylase messenger RNA in the surviving dopamine neurons of the substantia nigra in Parkinson’s disease: an in situ hybridization study. Neuroscience 38, 245–253 10.1016/0306-4522(90)90389-L 1979431

[B38] Jones B. E. Moore R. Y. (1977). Ascending projections of the locus coeruleus in the rat. II. Autoradiographic study. Brain Res. 127, 23–53 10.1016/0006-8993(77)90377-8 301051

[B39] Jones B. E. Yang T. Z. (1985). The efferent projections from the reticular formation and the locus coeruleus studied by anterograde and retrograde axonal transport in the rat. J. Comp. Neurol. 242, 56–92 10.1002/cne.902420105 2416786

[B40] Jonsson G. Hallman H. Ponzio F. Ross S. (1981). DSP4 (N-(2-chloroethyl)-N-ethyl-2-bromobenzylamine)- a useful denervation tool for central and peripheral noradrenaline neurons. Eur. J. Pharmacol. 72, 173–188 10.1016/0014-2999(81)90272-7 6265244

[B41] Kask A. Harro J. Tuomainen P. Rago L. Mannisto P. T. (1997). Overflow of noradrenaline and dopamine in frontal cortex after [N-(2-chloroethyl)-N-ethyl-2-bromobenzylamine] (DSP-4) treatment: in vivo microdialysis study in anaesthetized rats. Naunyn Schmiedebergs Arch. Pharmacol. 355, 267–272 10.1007/PL00004942 9050022

[B42] Kastner A. Hirsch E. C. Herrero M. T. Javoy-Agid F. Agid Y. (1993). Immunocytochemical quantification of tyrosine hydroxylase in the mesencephalon of control subjects and patients with Parkinson’s and Alzheimer’s disease. J. Neurochem. 61, 1024–1034 10.1111/j.1471-4159.1993.tb03616.x 8103078

[B43] Lategan A. J. Marien M. R. Colpaert F. C. (1990). Effects of locus coeruleus lesions on the release of endogenous dopamine in the rat nucleus accumbens and caudate nucleus as determined by intracerebral microdialysis. Brain Res. 523, 134–138 10.1016/0006-8993(90)91646-X 1698514

[B44] Lategan A. J. Marien M. R. Colpaert F. C. (1992). Suppression of nigrostriatal and mesolimbic release in vivo following noradrenaline depletion by DSP-4: a microdialysis study. Life Sci. 50, 995–999 10.1016/0024-3205(92)90093-5 1372673

[B45] Lyons W. E. Fritschy J. M. Grzanna R. (1989). The noradrenergic neurotoxin DSP-4 eliminates the coeruleospinal projection but spares projections of the A5 and A7 groups to the ventral horn of the rat spinal cord. J. Neurosci. 9, 1481–1489 2542474 10.1523/JNEUROSCI.09-05-01481.1989 PMC6569829

[B46] MacRae-Degueurce A. Milon H. (1983). Serotonin and dopamine afferents to the rat locus coeruleus: a biochemical study after lesioning of the ventral mesencephalic tegmental-A10 region and the raphe dorsalis. Brain Res. 263, 344–347 10.1016/0006-8993(83)90327-X 6301650

[B47] Marien M. Briley M. Colpaert F. (1993). Noradrenaline depletion exacerbates MPTP-induced striatal dopamine loss in mice. Eur. J. Pharmacol. 236, 487–489 10.1016/0014-2999(93)90489-5 7689466

[B48] Matsukawa M. Nakadate K. Ishihara I. Okado N. (2003). Synaptic loss following depletion of noradrenaline and/or serotonin in the rat visual cortex: a quantitative electron microscopic study. Neuroscience 122, 627–635 10.1016/j.neuroscience.2003.08.047 14622906

[B49] Mavridis M. Degryse A.-D. Lategan A. J. Marien A. J. Colpaert F. C. (1991). Effects of locus coeruleus lesions on parkinsonian signs, striatal dopamine and substantia nigra cell loss after 1-methyl-4-phenyl-1,2,3,6-tetrahydropyridine in monkeys: a possible role for the locus coeruleus in the progression of Parkinson’s disease. Neuroscience 41, 507–523 10.1016/0306-4522(91)90345-O 1870701

[B50] McMahon A. Geertman R. Sabban E. L. (1990). Rat dopamine β-hydroxylase: molecular cloning and characterization of the cDNA and regulation of the mRNA by reserpine. J. Neurosci. Res. 25, 395–404 10.1002/jnr.490250317 2325165

[B51] McMillan P. J. White S. S. Franklin A. Greenup J. L. Leverenz J. B. Raskind M. A. (2011). Differential response of the central noradrenergic nervous system to the loss of locus coeruleus neurons in Parkinson’s disease and Alzheimer’s disease. Brain Res. 1373, 240–252 10.1016/j.brainres.2010.12.015 21147074 PMC3038670

[B52] Miguelez C. Grandoso L. Ugedo L. (2011). Locus coeruleus and dorsal raphe neuron activity and response to acute antidepressant administration in a rat model of Parkinson’s disease. Int. J. Neuropsychopharmacol. 14, 187–200 10.1017/S146114571000043X 20426885

[B53] Nagatsu T. Sawada M. (2007). Biochemistry of postmortem brains in Parkinson’s disease: historical overview and future prospects. J. Neural Transm. Suppl. 72, 113–120 10.1007/978-3-211-73574-9_14 17982884

[B54] Patt S. Gerhard L. (1993). A golgi study of human locus coeruleus in normal brains and in Parkinson’s disease. Neuropathol. Appl. Neurobiol. 19, 519–523 10.1111/j.1365-2990.1993.tb00480.x 8121544

[B55] Perry T. L. Yong V. W. Jones K. Wall R. A. Clavier R. M. Foulks J. G. (1985). Effects of N-methyl-4-phenyl-1,2,3,6-tetrahydropyridine and its metabolite, N-methyl-4-phenylpyridinium ion, on dopaminergic nigrostriatal neurons in the mouse. Neurosci. Lett. 58, 321–326 10.1016/0304-3940(85)90074-6 3876525

[B56] Phillipson O. T. (1979). Afferent projections to the ventral tegmental area of Tsai and interfascicular nucleus: a horseradish peroxidase study in the rat. J. Comp. Neurol. 187, 117–144 10.1002/cne.901870106 489776

[B57] Ricaurte G. A. Langston J. W. DeLanney L. E. Irwin I. Peroutka S. J. Forno L. S. (1986). Fate of nigrostriatal neurons in young mature mice given 1-methyl-4-phenyl-1,2,3,6-tetrahydropyridine: a neurochemical and morphological reassessment. Brain Res. 376, 117–124 10.1016/0006-8993(86)90905-4 3487376

[B58] Robertson G. B. Fluharty S. J. Zigmond M. J. Sclabassi R. J. Berger T. W. (1993). Recovery of hippocampal dentate gyrus granule cell responsiveness to entorhinal cortical input following norepinephrine depletion. Brain Res. 61, 21–28 10.1016/0006-8993(93)91013-i 7688646

[B59] Rommelfanger K. S. Edwards G. L. Freeman K. G. Liles L. C. Miller G. W. Weinshenker D. (2007). Norepinephrine loss produces more profound motor deficits than MPTP treatment in mice. Proc. Natl. Acad. Sci. U.S.A. 104, 13804–13809 10.1073/pnas.0702753104 17702867 PMC1959463

[B60] Rommelfanger K. S. Weinshenker D. (2007). Norepinephrine: the redheaded stepchild of Parkinson’s disease. Biochem. Pharmacol. 74, 177–190 10.1016/j.bcp.2007.01.036 17416354

[B61] Ross S. B. (1976). Long-term effects of N-(2-chloroethyl)-N-ethyl-2-bromobenzylamine hydrochloride on noradrenergic neurons in the rat brain and heart. Br. J. Pharmacol. 58, 521–527 10.1111/j.1476-5381.1976.tb08619.x 1000130 PMC1667482

[B62] Sanders J. D. Szot P. Weinshenker D. Happe H. K. Bylund D. B. Murrin L. C. (2006). Analysis of brain adrenergic receptors in dopamine-β-hydroxylase knockout mice. Brain Res. 1109, 45–53 10.1016/j.brainres.2006.06.033 16854392

[B63] Simon H. Le Moal M. Calas A. (1979). Efferents and afferents of the ventral tegmental=A10 region studied after local injection of [3H]leucine and horseradish peroxidase. Brain Res. 178, 17–40 10.1016/0006-8993(79)90085-4 91413

[B64] Singh N. Pillay V. Choonara Y. E. (2007). Advances in the treatment of Parkinson’s disease. Prog. Neurobiol. 81, 29–44 10.1016/j.pneurobio.2006.11.009 17258379

[B65] Srinivasan J. Schmidt W. J. (2003). Potentiation of parkinsonian symptoms by depletion of locus coeruleus noradrenaline in 6-hydroxydopamine-induced partial degeneration of substantia nigra in rats. Eur. J. Pharmacol. 17, 2586–2592 10.1046/j.1460-9568.2003.02684.x 12823465

[B66] Srinivasan J. Schmidt W. J. (2004). Behavioral and neurochemical effects of noradrenergic depletions with N-(2-chloroethyl)-N-ethyl-2-bromobenzylamine in 6-hydroxydopamine-induced rat model of Parkinson’s disease. Behav. Brain Res. 151, 191–199 10.1016/j.bbr.2003.08.016 15084435

[B67] Swanson L. W. Hartman B. K. (1975). The central adrenergic system. An immunofluorescence study of the location of cell bodies and their efferent connections in the rat utilizing dopamine-β-hydroxylase as a marker. J. Comp. Neurol. 163, 467–505 10.1002/cne.901630406 1100685

[B68] Szot P. Knight L. Franklin A. Sikkema C. Foster S. Wilkinson C. W. (2012). Lesioning noradrenergic neurons of the locus coeruleus in C57Bl/6 mice with unilateral 6-hydroxydopamine injection, to assess molecular, electrophysiological and biochemical changes in noradrenergic signaling. Neuroscience 216, 143–157 10.1016/j.neuroscience.2012.04.046 22542679 PMC3383824

[B69] Szot P. Miguelez C. White S. S. Franklin A. Sikkema C. Wilkinson C. W. (2010). A comprehensive analysis of the effect of DSP4 on the locus coeruleus noradrenergic system in the rat. Neuroscience 166, 279–291 10.1016/j.neuroscience.2009.12.027 20045445 PMC4060967

[B70] Szot P. White S. S. Greenup J. L. Leverenz J. B. Peskind E. R. Raskind M. A. (2006). Compensatory changes in the noradrenergic nervous system in the locus coeruleus and hippocampus of postmortem subjects with Alzheimer’s disease and dementia with Lewy bodies. J. Neurosci. 26, 467–478 10.1523/JNEUROSCI.4265-05.2006 16407544 PMC6674412

[B71] Szot P. White S. S. Veith R. C. (1997). Effect of pentylenetetrazol on the expression of tyrosine hydroxylase mRNA and norepinephrine and dopamine transporter mRNA. Brain Res. Mol. Brain Res. 44, 46–54 10.1016/S0169-328X(96)00217-3 9030697

[B72] Taylor T. N. Caudle W. M. Sheperd K. R. Noorian A. Jackson C. R. Iuvone P. M. (2009). Nonmotor symptoms of Parkinson’s disease revealed in an animal model with reduced monoamine storage capacity. J. Neurosci. 29, 8103–8113 10.1523/JNEUROSCI.2217-09.2009 19553450 PMC2813143

[B73] Theron C. N. de Villiers A. S. Taljaard J. J. (1993). Effects of DSP-4 on monoamine and monoamine metabolites levels and on beta adrenoreceptor binding kinetics in rat brain at different times after administration. Neurochem. Res. 18, 1321–1327 10.1007/BF00975054 7505893

[B74] Thomas S. A. Marck B. T. Palmiter R. D. Matsumoto A. (1998). Restoration of norepinephrine and reversal of phenotypes in mice lacking dopamine-β-hydroxylase. J. Neurochem. 70, 2468–2476 10.1046/j.1471-4159.1998.70062468.x 9603211

[B75] Wang Y. Zhang Q. J. Liu J. Ali U. Gui Z. H. Hui Y. P. (2010). Noradrenergic lesion of the locus coeruleus increases apomorphine-induced circling behavior and the firing activity of substantia nigra pars reticulata neurons in a rat model of Parkinson’s disease. Brain Res. 1310, 189–199 10.1016/j.brainres.2009.10.070 19896932

[B76] Weinshenker D. White S. S. Javors M. A. Palmiter R. D. Szot P. (2002). Regulation of norepinephrine transporter abundance by catecholamines and desipramine in vivo. Brain Res. 946, 239–246 10.1016/S0006-8993(02)02889-5 12137927

[B77] Wolfman C. Abo V. Calvo D. Medina J. Dajas F. Silveiro R. (1994). Recovery of central noradrenergic neurons one year after the administration of the neurotoxin DSP4. Neurochem. Int. 25, 395–400 10.1016/0197-0186(94)90147-3 7820072

[B78] Zarow C. Lyness S. A. Mortimer J. A. Chui H. C. (2003). Neuronal loss is greater in the locus coeruleus than nucleus basalis and substantia nigra in Alzheimer’ and Parkinson disease. Arch. Neurol. 60, 337–341 10.1001/archneur.60.3.337 12633144

